# P-1880. An Innovative Infectious Diseases Hospitalist Model - Supporting Lean ID Teams

**DOI:** 10.1093/ofid/ofae631.2041

**Published:** 2025-01-29

**Authors:** Frances V Ue, Debjani Banerji, Lou Ann Bruno-Murtha

**Affiliations:** Massachusetts General Hospital and Brigham and Women's Hospital, Cambridge Health Alliance, Harvard Medical School, Cambridge, Massachusetts; Cambridge Health Alliance/ Harvard Medical School, Cambridge, Massachusetts; Cambridge Health Alliance, Cambridge, MA

## Abstract

**Background:**

Two-thirds of the US population live in counties with no or below-average ID physician (ID/P) coverage. This workforce shortage is exacerbated by emerging ID threats and an inadequate fellowship match. In response to these demands, we developed an innovative ID hospitalist (ID/H) model. Subspecialty hospitalists have been increasing since the early 2000s, and ID/Hs may provide support to a stretched ID workforce and mitigate burnout.

**Figure:**
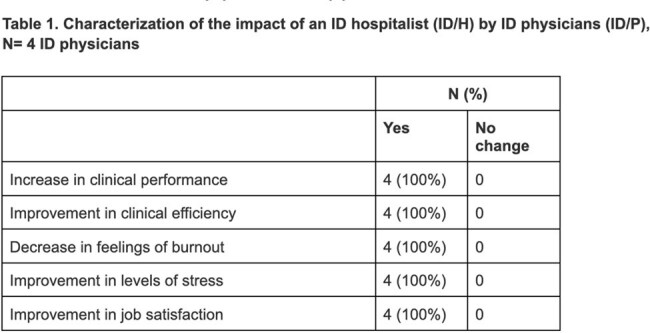

**Methods:**

Setting: Cambridge Health Alliance (CHA) is an academic, community, safety-net healthcare system in Massachusetts with two hospitals and multiple outpatient clinics. It serves >140,000 patients in Boston’s metro-north region.

Intervention: CHA’s ID Division is composed of 4 attending physicians (2 FTE total in ID), 1 physician’s assistant (PA), and 1 ID pharmacist. Inpatient consultations are usually performed by 2 ID/Ps (1 at each hospital campus) with partial PA support. During high volume and low staffing weeks from 7/2021 to 4/2023 with only 1 ID/P available, a new staffing model was created. An ID/H provided in-person consultations at one campus (with over-the-phone discussion with the ID/P), while the ID/P provided in-person consultations at the other campus. Outcomes were evaluated via surveys to the ID/Ps, chart review analyzing recommendations, and financial modeling of compensation rates at CHA and locum agencies.

**Figure:**
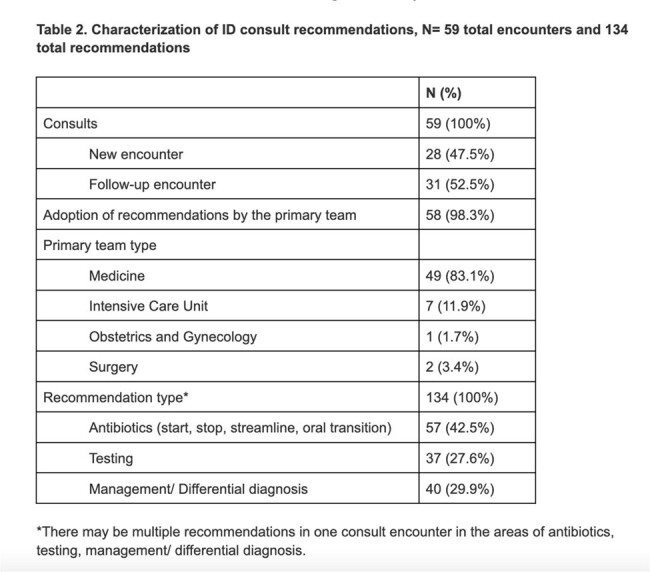

**Results:**

An ID/H completed 59 clinical encounters during 21 shifts over 9 weeks in the study period. All ID/Ps had high/highest satisfaction working with an ID/H when ranked on a Likert scale. ID/Ps characterized the impact of the ID/H in Table 1. Qualitative themes included an increase in staffing flexibility and timely patient care.

There was a high adoption rate of consult recommendations (58/59= 98.3%). The majority of consults were sought by the Medicine service (49/59= 83.1%). ID Consults are characterized in Table 2.

This model was cost-effective for department finances compared to utilization of ID moonlighters or locums. Locums are compensated at a much higher hourly rate and due to delays with insurance processing, the institution may not receive reimbursement for their work.

**Figure:**
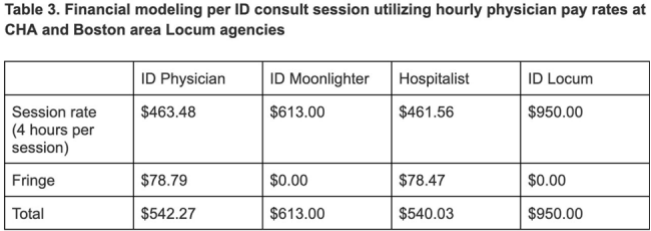

**Conclusion:**

An ID/H model is a novel way to support lean ID teams. Future research is needed to explore if this will attract more physicians to ID.

**Disclosures:**

All Authors: No reported disclosures

